# Optimization and Communication in UAV Networks

**DOI:** 10.3390/s20185036

**Published:** 2020-09-04

**Authors:** Christelle Caillouet, Nathalie Mitton

**Affiliations:** 1 Inria, 40 Avenue Halley, 59650 Villeneuve d’Ascq, France; 2 Department INFO of IUT Nice Côte d’Azur, Université Côte d’Azur, CNRS, I3S, 2004 Route des Lucioles, 06902 Sophia Antipolis, France

**Keywords:** UAV, drones, wireless, self-organization, optimization, swarm, communication, algorithms

## Abstract

Nowadays, Unmanned Aerial Vehicles (UAVs) have received growing popularity in the Internet-of-Things (IoT) which often deploys many sensors in a relatively wide region. Current trends focus on deployment of a single UAV or a swarm of it to generally map an area, perform surveillance, monitoring or rescue operations, collect data from ground sensors or various communicating devices, provide additional computing services close to data producers, etc. Applications are very diverse and call for different features or requirements. But UAV remain low-power battery powered devices that in addition to their mission, must fly and communicate. Thanks to wireless communications, they participate to mobile dynamic networks composed of UAV and ground sensors and thus many challenges have to be addressed to make UAV very efficient. And behind any UAV application, hides an optimization problem. There is still a criterion or multiple ones to optimize such as flying time, energy consumption, number of UAV, quantity of data to send/receive, etc.

## 1. Introduction

With new technological advances, UAVs are becoming a reality and are attracting more and more attention. UAVs or drones are flying devices that can be remotely controlled or, more recently, completely autonomous. They can be used alone or as a fleet, and in a large set of applications: from rescue operations to event coverage going through servicing other networks such as sensor networks for replacing, recharging, or data offloading. They are hardware-constrained since they cannot be too heavy and rely on batteries. Depending on their use (alone or in a swarm) and the targeted applications, they must evolve differently and meet different requirements (energy preservation, delay of covering an area, coverage, limited number of devices, etc.) with limited resources (energy, speed, etc.). Yet, their use still raises a large set of new exciting challenges, in terms of trajectory optimization, positioning, when they are used alone or in cooperation, coordination, and communication when they evolve in a swarm, just to name a few. This Special Issue was calling for any new original submissions that deal with UAV or UAV swarm optimization or communication aspects. Among the numerous submissions, only twelve of them have been selected after a rigorous selection process. The main themes that arise from them are: (i) ground data collection from the air, (ii) control of UAV swarm UAV-based Mobile Edge Computing and (iii) application-driven UAV based measurements.

In the following, we sum up the contributions of the papers published to this Special Issue for each category to then conclude by drawing future challenges and still open issues.

## 2. Ground Data Collection from the Air and Path Planning

It is becoming more and more common to imagine having data sensed from ground wireless sensors collected by UAV to alleviate wireless peer-to-peer communications between ground sensors and reduce their energy consumption. However, such a paradigm raises a set of new challenges such as how to prioritize the sensors to visit, how to optimize the time to collect all data by visiting all devices, etc. This is an exciting optimization problem. Works [1,2,3,4] propose different approaches to address this issue with different perspectives. Different criteria are considered to plan the trajectory of the UAV and different functions are optimized.

Reference [1] proposes to visit the nodes in a given order and for a variable time that depend on a node priority, while in [2], the authors aim to maximize the data collection utility by jointly optimizing the communication scheduling and trajectory of each UAV. The data collection utility is determined by the amount and value of the collected data and a novel trajectory planning algorithm is designed to maximize it. The author of [3] focuses on the problem of minimizing the mission completion time (flying time and hovering time) for a multi-UAV system in a monitoring scenario while ensuring that the information of each sensor is collected. As for [4], the authors aim to improve the secrecy performance of cellular-enabled unmanned aerial vehicle communication networks through an aerial cooperative jamming scheme.

## 3. Control of UAV Swarm

When more than a drone is required, a swarm of UAVs can be deployed. Although bringing more performances in terms of coverage and connectivity, new optimization challenges pop up due to the difficulty to control and scale such swarms both in a distributed or centralized way. References [5,6,7,8] tackle these numerous challenges going from connectivity maintenance to swarm control.

Reference [5] studies the different factors that may impact the accuracy and efficiency of an unmanned aerial vehicle (UAV) swarm coordination. The authors propose a mathematical data model to demonstrate the fundamental properties of antenna arrays and study the performance of the data collection system framework. Numerical examples and practical measurements are provided to demonstrate the feasibility of the proposed data collection system framework using an iterative-MUSIC algorithm and benchmark theoretical expectations.

Reference [6] deals with multi-UAV systems where the UAV autonomy is much smaller than the time to complete their mission. The authors thus introduce a UAV replacement procedure as a way to guarantee ground users’ connectivity over time, formulating the practical UAV replacements problem in moderately large multi-UAV swarms and proves it to be an NP-hard problem in which an optimal solution has exponential complexity. Reference [7] focuses on the maintenance formation with time-varying shape of a swarm proposing a virtual leader approach while [8] investigates a stochastic model of the UAV Swarm system with multiplicative noises.

## 4. UAV Enabled Mobile Edge Computing

The potential offered by the abundance of sensors, actuators, and communications in the Internet of Things (IoT) era is hindered by the limited computational capacity of local nodes. However, the latter do not necessarily always have the capacity to offload data to an edge server. In such a case, mobile edge servers can go to them thanks to the deployment of UAV-assisted Multi-access Edge Computing systems, which raises new challenging optimization and networking issues as addressed in [9,10].

Reference [9] proposes to provide an Unmanned Aerial Vehicle (UAV)-assisted Multi-access Edge Computing (MEC) system based on a usage-based pricing policy for allowing the exploitation of the servers’ computing resources while the authors of [10] introduce the DRUID-NET perspective, aiming to adapt to a rapidly varying demand by applying different tools from Automata and Graph theory, Machine Learning, Modern Control Theory, and Network Theory combined.

## 5. Application-Driven UAV Based Measurements

In such cases, the application that has asked for UAV deployment comes with very specific constraints and requirements and calls for specific optimization models. Reference [11,12] gives two such examples dedicated respectively for three-dimensional measurements and surveillance.

For instance, Reference [11] aims to provide a comparative analysis of the precision of ground geodetic data versus the three-dimensional measurements from unmanned aerial vehicles (UAV), while establishing the impact of herbaceous vegetation on the UAV 3D model. A constraint to take into account in this application is the fact that herbaceous vegetation can impede the establishment of the anthropogenic roughness of the surface and deteriorates the identification of minor surfaces.

Reference [12] focuses on UAV cooperative surveillance networks and introduces the use of complex field network coding (CFNC) for this application. According to whether there is a direct communication link between any source drone and the destination, the information transfer mechanism at the downlink is set to one of two modes, either mixed or relay transmission, and two corresponding irregular topology structures for CFNC-based networks are proposed. Theoretical analysis and simulation results with an additive white Gaussian noise (AWGN) channel show that the CFNC obtains a throughput as high as 1/2 symbol per source per channel use. Results show that CFNC applied to the proposed irregular structures under the two transmission modes can achieve better reliability due to full diversity gain as compared to that based on the regular structure.

## 6. Conclusions

As you can notice, challenges in UAV networks are huge, numerous and heterogeneous. They are concerning different aspects of the deployment of drones, from path trajectory to connectivity maintenance going through energy management. Much of them have been addressed with optimization tools but there remain a lot of open issues and research directions. Contributions presented in this special issue are only a first step to pave the way towards even more exciting investigations.

## References

[1] Ma X., Liu T., Liu S., Kacimi R., Dhaou R. (2020). Priority-Based Data Collection for UAV-Aided Mobile Sensor Network. Sensors.

[2] Qin Z., Dong C., Wang H., Li A., Dai H., Sun W., Xu Z. (2019). Trajectory Planning for Data Collection of Energy-Constrained Heterogeneous UAVs. Sensors.

[3] Qin Z., Li A., Dong C., Dai H., Xu Z. (2019). Completion Time Minimization for Multi-UAV Information Collection via Trajectory Planning. Sensors.

[4] Sun H., Duo B., Wang Z., Lin X., Gao C. (2019). Aerial Cooperative Jamming for Cellular-Enabled UAV Secure Communication Network: Joint Trajectory and Power Control Design. Sensors.

[5] Chen Z., Yeh S., Chamberland J.F., Huff G.H. (2019). A Sensor-Driven Analysis of Distributed Direction Finding Systems Based on UAV Swarms. Sensors.

[6] Sanchez-Aguero V., Valera F., Vidal I., Tipantuna C., Hesselbach X. (2020). Energy-Aware Management in Multi-UAV Deployments: Modelling and Strategies. Sensors.

[7] Cordeiro T.F.K., Ishihara J.Y., Ferreira H.C. (2020). A Decentralized Low-Chattering Sliding Mode Formation Flight Controller for a Swarm of UAVs. Sensors.

[8] Zhao H., Wu S., Wen Y., Liu W., Wu X. (2019). Modeling and Flight Experiments for Swarms of High Dynamic UAVs: A Stochastic Configuration Control System with Multiplicative Noises. Sensors.

[9] Mitsis G., Tsiropoulou E.E., Papavassiliou S. (2020). Data Offloading in UAV-Assisted Multi-Access Edge Computing Systems: A Resource-Based Pricing and User Risk-Awareness Approach. Sensors.

[10] Dechouniotis D., Athanasopoulos N., Leivadeas A., Mitton N., Jungers R., Papavassiliou S. (2020). Edge Computing Resource Allocation for Dynamic Networks: The DRUID-NET Vision and Perspective. Sensors.

[11] Cesnulevicius A., Bautrėnas A., Bevainis L., Ovodas D. (2019). A Comparison of the Influence of Vegetation Cover on the Precision of an UAV 3D Model and Ground Measurement Data for Archaeological Investigations: A Case Study of the Lepelionys Mound, Middle Lithuania. Sensors.

[12] Xue R., Han L., Chai H. (2020). Complex Field Network Coding for Multi-Source Multi-Relay Single-Destination UAV Cooperative Surveillance Networks. Sensors.

